# *"These Questions Have Everything That Happens to me"*: Analysis of a Femicide Risk Assessment Tool for Abused Women in Brazil

**DOI:** 10.1007/s10896-021-00313-1

**Published:** 2021-09-03

**Authors:** Dabney P. Evans, Casey D. Xavier Hall, Raiza Wallace Guimarães da Rocha, Sandra Marques Prado, Marcos C. Signorelli

**Affiliations:** 1 Hubert Department of Global Health, Rollins School of Public Health, Emory University, 1518 Clifton Road, NE, Atlanta, GA 30322 USA; 2 Department of Medical Social Sciences, Feinberg School of Medicine, Northwestern University Chicago, Chicago, IL USA; 3 Institute for Sexual and Gender Minority Health and Wellbeing, Northwestern University, Chicago, IL USA; 4 Chamber of Public Health, Federal University of Paraná, Paraná, Brazil; 5 House of the Brazilian Woman of Curitiba, Curitiba, Brazil

**Keywords:** Femicide, IPV, VAW, Violence, Brazil, Risk assessment

## Abstract

**Purpose:**

The purpose of this mixed-methods triangulation study was to assess the face validity and comprehension of a femicide risk assessment tool, the Danger Assessment-Brazil (DA-Brazil) among women seeking care in a one stop center for abused women in Curitiba, Brazil. Our secondary aim was to assess professionals' perceptions of feasibility for using the DA-Brazil in the same setting.

**Method:**

Fifty-five women experiencing relationship violence completed the instrument and participated in cognitive interviews about their experience; professionals attending survivors were also interviewed.

**Results:**

The vast majority of women described the DA-Brazil instrument as being easy to comprehend (*n* = 41, 73.2%). Nearly half of participants (*n* = 26, 46.4%) had some kind of question regarding the DA-Brazil calendar, a tool to visualize abuse frequency and severity. Queries aligned with five categories: recollection of dates, scale, relationship status, terminology, and discomfort. Professionals reported that the DA-Brazil instrument would support referral decision-making.

**Conclusion:**

The overall face validity and comprehension of the DA-Brazil appears to be high. The majority of challenges were around the calendar activity. Professional perceptions of the DA-Brazil suggest a high degree of feasibility for its use in Brazilian healthcare settings. In order for the DA-Brazil to effectively be administered with facilitated support there is a need for training on the best use of the instrument. Accurate assessment of femicide risk is critical in a country like Brazil with high rates of femicide. The DA-Brazil provides a valid assessment of femicide risk and has the potential to trigger early intervention for those at risk.

**Supplementary Information:**

The online version contains supplementary material available at 10.1007/s10896-021-00313-1.

## Background

Intimate femicide, the homicide of a woman by her current or former intimate partner is an extreme form of Intimate Partner Violence (IPV). In Brazil, four women are killed each day in this way. As a result, the country ranks 6^th^ in the world for femicide (Waiselfisz, 2015). Brazil’s rate of 4.8 female homicides per 100,000 women (Waiselfisz, 2015) is 2.5 times higher than the global average (Racovita, 2015), posing a significant public health problem as a result of mortality due to femicide. Brazil has responded to IPV and femicide through the passage of major federal laws, such as the Maria da Penha Law (Law No. 11.340/2006) (Brasil, 2006). In 2015 the country enacted an anti-femicide statute (Law No. 13.104) (Brasil, 2015) demonstrating the political will to prevent femicide at the national level (Gattegno et al., 2016).

Femicide prevention requires a multilevel approach including interprofessional support and institutional interventions to identify and report IPV before it escalates (Xavier Hall & Evans, 2020). Brazil institutionalized its commitment to preventing IPV and femicide through the 2015 establishment of the House of the Brazilian Woman (HBW) (Brasil, 2013). The HBWs are a national network of 24/7 public resource one stop centers (OSC) providing comprehensive services for people experiencing violence (Johnson, 2020; Olson et al., 2020). Seven HBW are currently in operation including our study site in Curitiba, Brazil (Curitiba, 2016). Despite the promise of these structures, impunity for perpetrators of violence (Evans et al., 2018), lack of trust in the health and legal sectors (Evans et al., 2020a, 2020b), barriers to addressing IPV among health providers (Evans et al., 2019), and other factors impede the prevention of IPV and femicide. A recent study revealed that the Curitiba’s Metropolitan area faces higher rates of femicide compared to the state average and the most common location of these crimes was in the home (35%) using firearms (44%) (Wanzinack et al., 2020).

In high-income countries, femicide prevention efforts have included the use of risk assessment tools. The Danger Assessment (DA) is a validated risk assessment tool designed to predict IPV recurrence as well as attempted and completed intimate femicide (for more detail on its structure and application see: Campbell et al., 2009; Weisz et al., 2000). The DA is used by women, law enforcement, courts and social service providers so that women and first responders may determine the need for protective action (Campbell, 2005). The DA has been periodically reviewed and updated according to specific needs, including adaptations for same-sex couples and immigrant populations in the US (Glass et al., 2008; Messing et al., 2013; Messing, Campbell, AbiNader, & Bolyard, 2020; Graham et al., 2021). The DA has been systematically translated for use in four languages. However, low- and middle-income countries (LMICs)—including those most affected by femicide like Brazil—have not yet benefited from these valuable tools. While the DA has been translated and adapted for use in a range of populations, no such assessment has been validated for use among Brazilians. Language differences, and the need for cultural adaption and rigorous validation act as barriers to the adoption of effective femicide risk strategies, such as the DA, in Brazil. This means that women who are at risk of femicide in Brazil are under identified, likely unaware of their risk, and not being connected to valuable services that could save their lives. Brazilian women, along with criminal justice and health professionals, need the creation of a culturally-appropriate risk assessment instrument like the DA to assist in identifying women who are at imminent risk of femicide in order to prevent it.

Using the cross-cultural framework developed by Gjersing and colleagues (Gjersing et al., 2010) and under the advice of the creator of the DA, the first author has translated the DA into Brazilian Portuguese and conducted a formative content evaluation of this translation among women in Brazil (Manders et al., 2021). The resulting instrument, the Danger Assessment-Brazil (DA-Brazil) is a cross-cultural adaptation and translation of the validated DA instrument (Manders et al., 2021) (See Annex 1). The DA-Brazil provides a culturally appropriate tool to assess femicide risk among Brazilian women, answering the call by the United Nations to prioritize the surveillance and prevention of femicide (UN News, 2020); however, it still requires further rigorous evaluation to ensure its validity in application. The cross-cultural framework necessitates that an adapted instrument be pre-tested to ensure that it is comprehended in the context where it will be applied (Gjersing et al., 2010). Some would also label the extent to which items are relevant, appropriate, and sensible to their intended audiences as *face validity*, which may also be impacted by the context in which the tool is applied (Fink, 2010; Holden, 2010). While face validity in itself does not ensure other forms of more technical forms of instrument validity it is an important aspect of measurement and screening that may impact participants understanding of an instrument as well as their emotional reactions to an instrument (Holden, 2010).

Despite the national response to femicide prevention in Brazil there is no systematic or validated way in which women’s femicide risk is assessed. As a result, the standardized and efficient identification of women most as risk for femicide, and thus, in need of intervention is absent. To address this critical need, the purpose of this mixed-methods triangulation study was to assess the face validity and comprehension of the DA-Brazil among women seeking support for IPV in a one stop center for abused women in Curitiba, Brazil. Our secondary aim was to assess professionals' perceptions on the feasibility of using the DA-Brazil in the same setting.

## Methods

### Study Site

The study took place in a one stop center for abused women, the House of the Brazilian Woman in Curitiba, Brazil (HBW-Curitiba), one of seven HBWs in operation nationally (Johnson et al., 2020). Since its founding in 2016 the HBW-Curitiba has provided comprehensive services to over 50,000 users (Almeida et al., 2020). Its services include health and legal services, shelter and temporary financial support. The HBW-Curitiba is staffed 24/7 by multidisciplinary teams focused on serving people experiencing physical, sexual, economic and/or psychological violence.

### Design

We utilized a triangulation approach (Minayo et al., 2005; Santos et al., 2020) to understand the complex nature of IPV, femicide and risk assessment. Our triangulation included (Santos et al., 2020): a) multiple methods including qualitative and quantitative approaches; b) triangulation of data, including DA-Brazil scores and cognitive interviews; c) various participant groups—namely survivors and professionals who work at the HBW-Curitiba; and d) triangulation of researchers and disciplines, including Brazilian and North American researchers, from diverse interdisciplinary backgrounds (human rights, public health and social sciences).

### Instrument

The DA-Brazil consists of a calendar and a set of twenty yes/no statements related to known femicide risk factors (Manders et al., 2021). The calendar is used to document the frequency of abuse; each incident on the calendar is also rated by its severity on a five-point scale. The calendar results in a visual of how often and serious violence within a given relationship is. The yes/no statements include statements related to strangulation, gun ownership, substance use, femicide and suicide threats, and other abusive behaviors.

### Participants

Upon arrival at the HBW-Curitiba women are evaluated by the HBW-Curitiba psychosocial staff to determine their service needs. Self-identified women over age 18 attending the HBW-Curitiba and deemed not to be in immediate crisis were eligible for participation. These women completed the DA-Brazil and participated in cognitive interviews about their experience with the instrument. We also interviewed professionals from the psychosocial support department who provide the first line of support to people attending the HBW. These participants were psychologists and social workers employed at the HBW-Curitiba.

### Data Collection

The DA-Brazil was presented to the psychosocial staff of the HBW-Curitiba in an informal orientation. Next, HBW-Curitiba psychosocial staff were asked to identify potential participants for the study during the data collection period (May–September 2020). Aiming for a diverse sample of participants, the study team administered a brief demographic survey (age, marital status, race/ethnicity, income, years of education, neighborhood, sexual orientation and gender identity), followed by self-administration of the DA-Brazil. Once finished each person was asked if they found the instrument difficult to complete and whether any terms were unclear; this session was audio recorded. Participants, all of whom self-identified as women, reported unclear terms to the research team, and they jointly discussed women’s queries and interpretations; the team probed to understand women’s face validity and comprehension of the DA-Brazil. For the purpose of this study, we considered face validity when an instrument measures what it is intended to measure, using appropriate language and language level to do so (Fink, 2010).

Additionally, interviews were conducted with HBW-Curitiba psychosocial staff (*n* = 4) in a private room during working hours. Initially, the study team presented the DA-Brazil and brief demographic survey. Professionals were asked about the DA-Brazil, their comprehension and concerns, and their reflections on women’s understanding of the instrument. Professionals also provided their perceptions about the feasibility of using the DA-Brazil as a routine risk assessment tool at the HBW-Curitiba. The recordings of the sessions (women and professionals) were transcribed verbatim in Portuguese.

### Data Analysis

Variables from the demographic survey were analyzed by frequency, percentage, mean and standard deviation when appropriate. Qualitative questions or comments made by women relating to each item, the calendar, and the overall ease of the instrument were extracted from the interviews. Questions or comments from professional’s perspectives were also extracted. For qualitative analysis we adopted thematic analysis of emerging categories, as described by Liamputtong (2009, 2010): first, reading each transcript and making sense of each interview, followed by a collective examination of what was being said by the participants as a group. Two authors (one being a native speaker of Portuguese, the other having studied Portuguese as a second language) independently translated these comments into English and manually coded these qualitative data into themes. If there was disagreement in the coding, a third author, also fluent in Portuguese reconciled the disagreement. For triangulation (Minayo et al., 2005; Oliveira et al., 2020), these different inputs (qualitative and quantitative), from different participant groups (women and professionals), were discussed and analyzed by the interdisciplinary research team for final analysis.

### Ethics

The study was approved by the Ethics Board Committees of the Federal University of Paraná and the City of Curitiba (CAAE approval number 89411818.4.0000.0102). The Emory University Institutional Review Board found the study to be exempt. Study staff utilized WHO guidelines (WHO, 2016) on conducting research on violence against women, and WHO ethical standards for research during public health emergencies (WHO, 2020) and deferred to the judgement of HBW-Curitiba psychosocial staff who advised which cases were suitable for inclusion in the study; people deemed to be in crises by the HBW-Curitiba psychosocial staff (*n* = 21) were excluded from our sample. All participants received the standard of care available at the HBW-Curitiba.

## Results

### Demographics

Fifty-six people (*n* = 56) self-identifying as abused women participated in the study. The majority of women came from Curitiba with only four (7.1%) coming from other cities in the metropolitan area (Almirante Tamandare, Colombo, Fazenda Rio Grande, and São José dos Pinhais) (Table 1). Women came from 32 different neighborhoods providing a diverse socio-economic profile. The majority of women (64.3%) reported a monthly income of R$1,040 to 4,159 (equivalent to $195 to $780 in the last quarter of 2020), considered a low to medium income level by Brazilian standards.

**Table 1:** Univariate analysis describing sample of women who reviewed the Danger Assessment (n = 56)

Variable	N (%)	Mean (SD)
Age		37.4 (10.1)
Race		
White	41 (73.2%)	
Multiracial	7 (12.5%)	
Black	6 (10.7%)	
Asian	2 (3.6%)	
Gender		
Cisgender	55 (98.2%)	
Transgender	1 (1.8%)	
Sexual Orientation		
Heterosexual	53 (94.6%)	
Bisexual	2 (3.6%)	
Lesbian	1 (1.8%)	
Disability Status		
None reported	52 (92.9%)	
Visual	2 (3.6%)	
Physical	1 (1.8%)	
Auditory	1 (1.8%)	
Relationship Status		
Single	18 (32.1%)	
Married	16 (28.6%)	
Partnered/civil union	14 (25.0%)	
Divorced or separated	8 (14.3%)	
Head of Household		
Yes	37 (66.1%)	
No	19 (33.9%)	
Number of Cohabitating Family Members		3.3 (1.4)
Family Income (in Brazilian Real R$)		
0 to 1039 (Low)	11 (19.6%)	
1040 to 4159 (Middle)	36 (64.3%)	
4160 to 10,400 (High)	7 (12.5%)	
Don’t know	2 (3.6%)	
Occupation		
Unemployed	22 (39.3%)	
Formally Employed	16 (28.6%)	
Self-employed	11 (19.6%)	
Government worker	3 (5.4%)	
Domestic worker/maid	4 (7.1%)	
Education		
Middle school or less	9 (16.1%)	
Some high school	6 (10.7%)	
High school	22 (39.3%)	
Some college	9 (16.1%)	
College degree	7 (12.5%)	
Graduate school	3 (5.4%)	
City		
Curitiba	52 (92.9%)	
Other	4 (7.1%)	
Danger Assessment Score		10.0 (3.9)
< = 7 “variable danger”	12 (21.4%)	
8–13 “increased danger”	37 (66.1%)	
14–17 “severe danger”	5 (8.9%)	
> = 18 “extreme danger”	2 (3.6%)	

Though the focus of interviews with women was not to gather information about their personal experiences of relationship violence, over the course of the interviews some women volunteered details of their experiences. Examples of experiences included persistent stalking, violence lasting years, husbands refusing divorce, the need for restraining orders, and fear of being murdered to name a few. Based on their DA-Brazil scores, nearly two thirds of women (66.1%) were in increased anger of femicide, with an additional 12.5 % being in either severe (8.9%) or extreme (3.6%) danger. These experiences  and preliminary scores reinforced the relevance and need for a risk assessment tool like the DA-Brazil.

Some queries and important concerns emerged from the interviews. We present more details on these concerns in the following sections on face validity and comprehension, the DA-Brazil calendar, women’s perceptions of the DA-Brazil and professionals’ perspectives about the feasibility of using the DA-Brazil.

### DA-Brazil: Face Validity and Comprehension Among Women

Five participants (8.9%) asked the researcher to read the questionnaire for them, because they were too nervous to read it. While this was a minority of women, it does highlight the importance of the mode of administration, and trauma-informed practices when implementing risk assessments. About forty percent of participants (*n* = 22) reported no difficulties in filling out the DA-Brazil or comprehension issues. The vast majority of women described the DA-Brazil instrument as being easy to comprehend (*n* = 41, 73.2%). Only one participant (1.8%) described the instrument as difficult to understand. Among the 21 instrument items, fourteen (66.7%) items did not raise any questions. One participant shared “I found it easy to answer. These questions have everything that happens to me” (W4). However, most participants (*n* = 33, 58.9%) had at least some query when completing the DA-Brazil. The majority of the questions were in regard to the calendar portion of the instrument.

### DA-Brazil Calendar: Women’s Perspectives

The calendar portion of the DA-Brazil appeared to be the most challenging aspect of the instrument for Brazilian women. The purpose of the calendar is for women to mark the dates when abuse happened during the preceding twelve months, specifying the degree of severity, ranging from 1 to 5 points, similar to a Likert Scale. Nearly half of participants (*n* = 26, 46.4%) had some kind of question regarding this aspect of the instrument. These were aligned with five general categories: recollection of dates, the calendar scale, relationship status, terminology, and discomfort in answering some questions.

In terms of recalling the specific dates when violence or injury occurred, participants discussed difficulty knowing the exact date when instances occurred and how to mark these experiences on the calendar. One participant shared,

*“I found it difficult, because when an aggression occurs, we will not remember exactly the day or how it happened...there will always be a reason for fights. Everything I could remember I put. Not all threats...not all attacks.”(W15, 26 years old).*

One participant who ended her relationship because her boyfriend frequently humiliated her, threatened to kill her dog and to share intimate pictures with her boss, revealed: *“And if I don't remember the day exactly, can I guess the month? I had to mark it on the calendar or here on the sheet* [of the instrument]*?”* (W64, age 27). Participants also discussed concern about experiences that were recurring or occurred over the course of consecutive days. They reported it was difficult to scale the abuse according to the items, because many types of abuses were concurrent. One participant whose husband frequently beat her and had previously attempted to kill her said: *“What if it is the case of* [having suffered] *all these* [items] *here, from the first to the fourth?”* (W 13, age 41) Participants appeared unclear about how to correctly complete the calendar and score the abuse, because different types of abuse happened simultaneously and over a long period of time.

Participants also raised concerns about how their relationship status may be confusing when completing the calendar, because some of them are in a current relationship, however the perpetrator is a former partner and not the current one. The calendar is intended to cover the prior 12 months, but in some reported cases, participants were separated from their former partner for more than a year at the time of filling out the instrument, and some were engaged in new relationships; even so, former partners continued abusing them. This was the case of a 25-year-old participant who is currently married but who is still at risk of violence from her ex-husband, who constantly threatens to kill her. “*How do I answer this? I broke up with him over a year ago. Do I need to fill this out* [calendar]?” (W 76, age 25). The confusion about which partner to refer to was challenging the women in this situation, particularly among those experiencing a prolonged threat from a former partner.

Some women raised concerns about the terminology adopted in the calendar section, that may not reflect the lived experiences of abuse. A divorced participant was constantly threatened during the pandemic by her former partner, who wanted visitation with their son who is at high-risk of COVID-19 infection. When she denied his requests because of COVID-19 exposure concerns he physically hurt her. She revealed:

*“Oh, is it to mark which of these happened to me? So, I mark here the two times he tried to hit me drunk, he tried to tear my clothes and stuff. And once he had drunk too and tried to squeeze my finger like that* [makes a gesture of how the finger was squeezed]*, but it didn't hurt, it just got swollen* [she smiles]*. I even said to him "you will break my finger...you will see." Because we were fighting and he was drunk. Then what do I write? Because it was not like this, slap and punch as it is written here****.****”* (W 4, age 23).

This participant was referring to the scale included in the calendar section, which includes: “1. Slapping, pushing; no injuries and/or lasting pain” and “2. Punching, kicking; bruises, cuts, and/or continuing pain” among others. As the abuses she suffered, including squeezing her finger, were not exactly mentioned in this scale, she was unsure about how she should respond. Others reported that the stress of their situation made it difficult to complete the calendar. A married participant who had been kicked out of her home shared, *“I didn't quite understand it here. I don't know if it is, because I'm nervous. My psyche is very affected. Ah! I didn't suffer any of that. Only curses and psychological violence”* (W 36, age 42). Terminology may also obscure other abuses they are experiencing, including those not specifically mentioned in the instrument, but that may offer potential risk. One participant described a clear pattern of coercive control and violence in her past relationship, but also described under-reporting her experiences, because she prefers to move on from past negative experiences.

*“Sometimes when we are in a relationship with a person who uses alcohol and drugs, the person is altered...like in my case, the person thinks that he is the king of the world, that he is right. Then I'm at home working, anything is a matter of discussion. I, who am a woman, do everything to avoid. But for the man, it's not cool, nothing is good ... I prefer not to remember too much, otherwise I'm going back to the past ... all that ... As I decided to take the initiative to come here today, I prefer to leave everything in the past and move on.” (W 15, age 26)*

### DA-Brazil Instrument: Women’s Perspectives

Nearly a quarter of participants (*n* = 13, 23.2%) had general questions or comments about the DA-Brazil instrument. These included the need for an explanation of how to complete the instrument, hesitancy to discuss the topic of violence, and sensitization on different forms of abuse. For example, one participant shared how the instrument itself raised her awareness about different types of violence,

“*I found it very easy. Here are several questions that I didn't think about when I came here. Because physical and verbal violence have really increased. Especially verbal. I think that verbal has been one of the most...frequent factors*” (W 7).

Only 7 out of 23 DA-Brazil instrument items (30.4%) prompted questions or concerns from the participants. These concerns centered on word choice and clarity (Table 2).

**Table 2:** Danger Assessment: Items, Questions and Comments About the Instrument from Brazilian Women

Danger Aassessment Questions^1^	Had question/comments		Summary of questions/comments (n)	Exemplary Quotes
	Yes n (%)	No n (%)		
1. Has the physical violence increased in severity or frequency over the past year? *1. A violência física tem aumentado em gravidade ou frequência no último ano?*	3 (5.4%)	53 (94.6%)	Confusion over the term “physical violence.” (2) Confusion due to timing/past year. (1)	“Is physical violence hitting? Isn’t it?” (W 84)
2. Does he own a gun? *2. Ele tem um revólver?*	1 (1.8%)	55 (98.2%)	Confusion over the term for gun (*revolver* versus *arma*). (1)	“This one about having a gun, I don't know.” (W 68)
3. Have you left him any time after living together during the past year? *3. Alguma vez no último ano você deixou de viver com ele depois de morarem juntos?*	6 (10.7%)	50 (89.1%)	Confusion about the meaning of living together due to time frame (4) Confusion whether to mark (1) Didn’t understand (1)	“I didn't understand the question if I stopped living with him in the last year. Like stop living together, but continued dating? This I answer in a snicker, there is no space to do here.” (W 60)
4. Is he unemployed? *4. Ele está desempregado?*	2 (3.6%)	54 (96.4%)	Not knowing, not having current contact (2)	“Here when you ask if he is unemployed I don't know, I don't know how his life is anymore. Can I put I don't know?” (W 4)
5. Has he ever used a weapon against you or threatened you with a lethal weapon? *5. Ele alguma vez usou uma arma contra você ou ameaçou você com uma arma ou algum objeto que poderia te matar?*	2 (3.6%)	54 (96.4%)	Confusion over the double-barreled question (1) and whether this includes general verbal threats in addition to physical threats. (1)	“There's a threat, but it's not with a gun or anything. Threat only with words. He threatens that because I left home and now I am with someone else, that I already know what my ending will be. That where he finds me he will kill me.” (W 7)
6. Does he threaten to kill you? *6. Ele ameaça matar você?*	1 (1.8%)	55 (98.2%)	Questions about implied killing vs. using the term “kill.” (1)	“He doesn't speak with those words that he is going to kill me. But he says he'll pass the car over me.” (W 7)
11. Does he use illegal drugs? By drugs, I mean “uppers” or amphetamines, “meth”, speed, angel dust, cocaine, “crack”, street drugs or mixtures *11. Ele usa drogas? Por drogas quero dizer anfetaminas, metanfetamina (cristal, glass, bola), cocaína ou outras*	3 (5.4%)	53 (94.6%)	Confusion over the type of drug, specifically if cannibis or marijuana is included. (3)	“He doesn't use any of these drugs, he uses marijuana…where do I put it?” (W 9)

In regard to word choice three questions arose. One question was a single instance on an individual item meaning that this concern was not duplicated across more than one participant. The question was in regard to item 2, which uses the Brazilian Portuguese term *revólve*r (revolver), which can be interchangeable with the Brazilian Portuguese term *arma* (gun). The participant asked to clarify this terminology. A second concern around the definition of violence was raised by two participants, but in slightly different ways. Item 1 uses the term *violência* “violence” instead of behavioral examples of violence. One participant asked if hitting is considered “violence”, while another mentioned threats. Without behavioral examples participants are left to interpret the term “violence.” These comments represented small clarifications that were not common across the sample of women. Three women asked a question about item 11 which is about *drogas* “drug” use, but does not list cannabis. Because it was not listed in the examples provided by DA-Brazil the participants were unsure if cannabis should be considered a drug.

Several questions about clarity arose; similar to comments about word choice there were no consistent patterns across women or items. Item 5 inquires about threats with a weapon. Participant 7 asked about threats on life that do not involve an explicit weapon and whether these should be included in the response to this item. Another inquiry about threats was in regard to item 1, which addresses increases in severity of violence. Participant 4 asked *“Here when you ask if the severity of the violence has increased, I write no, right? Because he has only threatened,”* highlighting that threats—even increasing threats—may not be seen as increasing severity of violence. Lastly, another comment paralleled concerns from the calendar activity which addressed temporality. Two participants asked about item 3, which discusses ending cohabitation. Participant 46 said, *“This one I didn't understand ‘did you ever stop living with him in the last year?,’ I haven't lived with him for 13 years. What do I score?”* (W 46, 39 years old). Participant 60 also wanted to clarify if this item included relationships that end when cohabitation ended. Some participants considered only the literal terms mentioned in the DA-Brazil, and thus, not inferring variations or similar situations that could also bring risk of femicide. A clear example was revealed by participant W 7, age 49, regarding the question "Does he threaten to kill you?" She stated, *“He doesn't speak with those words that he is going to kill me. But he says he'll pass the car over me.”* This difficulty in interpretation of some questions by some participants may be related to the generalized low education levels present in Brazilian society compared to other Latin American countries and high-income countries (Bruns et al., 2012). This may make the self-administration of the DA-Brazil challenging for some Brazilian women, requiring assistance in these cases. Overall, the questions in regard to the DA-scale were relatively infrequent, minor, and lacked any consistent pattern. These findings taken together seem to suggest a high degree of clarity, and minimal if any needed changes to the DA-Brazil instrument.

### DA-Brazil Instrument: Professional Perspectives

Professional members of the HBW psychosocial support team (*n* = 4) also provided their perspectives on the DA-Brazil. The team of psychologists and social workers provide counseling, information and referral services. Their perceptions were necessary to assess the feasibility of the DA-Brazil for use as a tool supporting their work. The professional participants identified potential benefits and challenges using the DA-Brazil in a specialized one stop center for abused women. As potential benefits, they reported that the calendar could help women to view their abuse episodes on a continuum and that the DA-Brazil instrument items would provide details on women’s experiences to support referral decision-making (Table 3). As challenges, they expressed the belief that some women could not self-administer the tool due to their heightened emotional state and/or the need for guidance on how to complete the instrument. Regarding the DA-Brazil item on drugs, professionals suggested adding crack cocaine to the list of examples, because this drug is frequently present in their patients’ narratives (cocaine is listed on the instrument but crack is not mentioned by name). They recommended that the DA-Brazil be administered after the initial provision of psychosocial care, once trust and safety has been established.

**Table 3:** Perceived Benefits and Challenges of using the Danger Assessment-Brazil Among Professionals

Benefits	“I found this calendar interesting. This is very good, because women get lost in time. For example: How long have you not seen each other? Then they answer: A month ago. And suddenly they start to report something that happened last week. So I found this idea of time very interesting for them.” (P 1)
	“I think these instruments are interesting to apply here [at the HBW], because they can help us to understand more elements about the cases. I suggest that the instruments have an identification number of medical records equal to that of our records. So that we know what is the case, especially if we need to activate another service. Their language is generally accessible. Eventually, if they have any questions, they can ask us.” (P 2)
	“This sentence here from the instrument about being mine and nobody else, is very recurrent here. Sometimes they are years apart, but the guy doesn't accept the separation. I think the language is good. It is adequate according to what they usually report. If she is going to fill it out and then she can ask questions with us…it's easy. I think that maybe filling it out already starts to realize the violence…If she answers the instrument before, maybe she will arrive at the service more organized.” (P 3)
Concerns	“Some cannot [manage to answer alone]. Of course, it depends on the case. Some come emotionally upset, so sometimes we have to repeat the explanation to them. About drugs, I think crack was missing here, which is very common here.” (P 1)
	“I was confused here: if you were strangled, passed out, etc. also write the letter E along with the number 4. Only with the number 4? Or can it, for example, associate E with 1, 2, 3, etc.? You may need to explain better here that the letter E can be associated with any number.” (P 1)
	“I think that the instrument needs to be delivered only after psychosocial care. Because they come with a certain expectation here, then we give guidance on whether or not it fits the Maria da Penha law. They arrive apprehensive. After our service they are better placed and would be able to answer the instrument with more tranquility. Even because it takes a long time between our service and the police station. So, in the meantime they can answer the instrument…They can stay in a private room and fill it out.” (P 2)

## Discussion

This study provides an analysis of triangulated data to examine face validity, comprehension, and feasibility for the use of the DA-Brazil, a culturally adapted translation of the Danger Assessment femicide risk assessment. The findings from this study provide an idea of overall face validity, an understanding of challenges, and evidence of feasibility for implementation in social and healthcare settings in Brazil.

The overall face validity, comprehension and ease of use of the DA-scale items appears to be high. Women expressed a high degree of perceived ease of use; there were a low number of questions and concerns about the instrument as well as few questions about individual survey items. The concerns that were raised centered around participant recall, and the timing of violent events relative to relationship status. In particular, several women reported experiencing ongoing harassment at the hands of partners from whom they had been separated more than one year. The intent of the original DA is to assess acute femicide risk, therefore issues related to ongoing psychological or emotional violence fall beyond its scope. However, the DA-Brazil appeared to sensitize participants to differing forms of violence —physical, sexual, psychological —which is in fact one of the secondary uses of the instrument (Campbell et al., 2009). In this way the instrument also acts as an intervention, raising awareness to violence that may otherwise have been normalized in the context of particular relationships. Overall, the finding that the DA-Brazil was easy to use and appropriate for most participants suggests that the high level of rigor used in translating and adapting the DA-Brazil was a worthwhile endeavor (Manders et al., 2021).

The majority of challenges relating to the DA-Brazil centered around the calendar activity. The calendar exercise is intended to assist women in mapping the frequency and severity of relationship violence over the prior 12 months; it includes a five-point scale, akin to a Likert scale and accompanying injuries (Manders et al., 2021). The calendar section of the DA was conceptualized as a way to raise consciousness about violence and reduce the normalization of IPV (Campbell, 1995). In the original DA development with US women, 38% of those who initially reported no increase in severity and frequency of physical violence in the past year altered their response to yes, after filling out the calendar section of the DA (Campbell, 1986, 1995). The challenges women experienced in self-administering the calendar exercise suggest that rather than marking the exact days when abuse happened (a concern revealed by some women), it may be adequate to mark approximately when abuse occurs which still reflects the frequency and escalation of abusive episodes. In order to assess ease of use, participants in our study engaged in a “cold read” of the DA-Brazil with minimal instructions on how to complete the calendar and survey instrument. As a result of our work, we were able to discern that the DA-Brazil calendar exercise requires explicit instructions if self-administered — or that the DA-Brazil should be administered in cooperation with trained professionals. Women’s difficulty in interpreting some parts of the instrument may be related to the generalized low education levels present in Brazilian society making self-administration of the DA-Brazil challenging for some women. This finding is consistent with the use of the DA in the US settings where women may complete the DA by themselves or with professional support from the health care, justice, or advocates who assist in interpreting the instrument scores (Campbell, 2005; Campbell et al., 2009).

Finally, professional perceptions of the DA-Brazil suggest a high degree of feasibility for its use in Brazilian social service health care settings. Professionals considered the DA-Brazil useful in supporting their work and improved accuracy in women’s disclosures of violence. Professionals perceived that using the DA-Brazil, women could remember incidents that might otherwise have gone undocumented. In order for the DA-Brazil to effectively be administered with facilitated support there is a need for training of professionals on the best use of the instrument, and the calendar in particular. Given that training materials and certification for use of the DA is available in English, these materials may be adapted for use with the DA-Brazil (Danger Assessment, n.d.). Professionals especially saw a use for the DA-Brazil in one stop centers survivors like the HBW-Curitiba. They believed that if completed prior to psychosocial intakes the instrument could guide conversations on safety planning in parallel with other assessments and tools (Campbell & Glass, 2009; Yaxley et al., 2018).

Accurate assessment of femicide risk is critical in a country like Brazil with high rates of IPV and femicide (Bailey et al., 1997; Waiselfisz, 2015); the DA-Brazil provides a valid assessment of femicide risk and has the potential to trigger early intervention for such cases. Future research on the use of the DA-Brazil may include a randomized control trial to assess the effectiveness of the instrument in triggering support mechanisms and preventing femicide relative to the standard of care.

## Limitations

Data collection for this study occurred in the context of the COVID-19 pandemic. COVID-19 has been associated with increased IPV and femicide including in Brazil (Roesch et al., 2020; Evans, Hawk & Ripkey, 2020; Marques et al., 2020; Scalzaretto, 2020). Although movement restrictions were in place in Curitiba, Brazil during our data collection period, the HBW remained open; the HBW was operating with reduced staffing, social distancing protocols and hygiene measures. It is possible that some women experiencing violence may have deferred seeking care because of the pandemic, though we would not expect them to differ substantially from those who did seek care.

Some women (*n* = 21) were excluded from our sample by HBW psychosocial staff who deemed these women to be in crisis. While we cannot be sure, we would expect that those who were excluded from our sample would have had higher scores on the DA-Brazil given their crisis designation. This means that our DA-Brazil scoring data are likely an underestimation of the spread or range of DA-Brazil scores and cannot be generalized; however, two women’s scores on the DA-Brazil indicated that they were in “extreme danger” underscoring the subjective nature of existing protocols. While this examination sets up a future randomized control trial of the DA-Brazil instrument it was not designed to fully validate the measure; other components of validity such as construct validity, criterion validity, and content validity were not assessed. However, because the risk factors for femicide are viewed as universal — and the original Danger Assessment is grounded in these risk factors —we believe that the tool is sufficiently ready for use in the Brazilian context.

## Conclusion

This analysis was focused on face validity and comprehension of the DA-Brazil instrument among Brazilian women experiencing IPV. We also sought to understand professionals’ perceptions about the feasibility of using this instrument in health care settings, specifically one stop centers for survivors of violence. Our triangulated approach garnering information from both women and professionals enabled us to minimize bias from a single analytical perspective. Overall, the face validity and comprehension of the instrument among women was high. Professionals believed the instrument had value and its use was feasible. We relied on previous validation studies of the Danger Assessment as well as our own work translating, cross culturally adapting the DA-Brazil (Campbell et al., 2009; Manders et al., 2021). This study on face validity and comprehension confirms that the DA-Brazil is a valuable risk assessment tool for identifying Brazilian women at high risk of femicide that could immediately be implemented in health care settings and social service settings.

## Supplementary Information

Below is the link to the electronic supplementary material.

Supplementary file1 (DOCX 27 KB)
