# Supplementary material for: "These Questions Have Everything That Happens to me": Analysis of a Femicide Risk Assessment Tool for Abused Women in Brazil
Source: J Fam Violence. 2021 Sep 3;37(4):547–57. doi: 10.1007/s10896-021-00313-1 (PMC8414033; doi:10.1007/s10896-021-00313-1)
Supplement: Supplementary file 1 — Supplementary file1 (DOCX 27 KB) [file 10896_2021_313_MOESM1_ESM.docx]

**AVALIAÇÃO DE RISCO**

Jacquelyn C. Campbell, PhD, RN. • Copyright, 2003, 2019 • [www.dangerassessment.org](http://www.dangerassessment.org)

Versão traduzida e adaptada com autorização da autora por Fonseca, Manita, Saavedra, & Magalhães (2013) versão para investigação • Versão adaptada ao português brasileiro por Manders, Vertamatti & Evans (2020).

Vários fatores de risco têm sido associados com aumento do risco de homicídio (assassinato) de homens e mulheres em relações violentas. Não podemos prever o que vai acontecer em seu caso, mas gostaríamos que você tivesse consciência do perigo de homicídio em situações de abuso e que você veja quantos fatores de risco se aplicam à sua situação.

Usando o calendário, por favor marque as datas aproximadas durante o último ano nas quais você sofreu abuso por parte do seu parceiro ou ex-parceiro. Escreva nesta data a gravidade do incidente ocorrido utilizando a seguinte escala:

1. Tapas, empurrões; sem machucados e/ou dor que dura
2. Murros, chutes; manchas roxas, cortes e/ou dor que dura
3. Surra; machucados graves, queimaduras, ossos quebrados
4. Ameaça de usar armas (armas brancas ou de fogo); machucados na cabeça, machucados internos, machucados permanentes, sofreu tentativa de estrangulamento/esganadura ou sofreu aborto. Se você foi estrangulada/ esganada e desmaiou (ou ficou tonta e/ou com a visão turva/escureceu a vista), também escreva letra “E” junto com o número 4. (Exemplo: 4E)
5. Uso de armas (armas brancas ou de fogo); feridas causadas por armas

(Se mais do que uma das descrições se aplica, escolha o número mais alto)

Responda "Sim" ou "Não" a cada uma das seguintes questões. ("Ele" refere-se ao seu marido, parceiro, ex-marido, ex-parceiro ou quem seja que atualmente está machucando você fisicamente.)

| ____ | 1. A violência física tem aumentado em gravidade ou frequência no último ano? | | |
| --- | --- | --- | --- |
| ____ | 2. Ele tem um revólver? | | |
| ____ | 3. Alguma vez no último ano você deixou de viver com ele depois de morarem juntos? | | |
|  |  | 3a. (Se nunca viveu junto com ele, marque aqui: __). |  |
| ____ | 4. Ele está desempregado? | | |
| ____ | 5. Ele alguma vez usou uma arma contra você ou ameaçou você com um objeto que poderia te matar? | | |
|  | (Se sim, a arma era um revolver? Marque aquí ___). | | |
| ____ | 6. Ele ameaça matar você? | | |
| ____ | 7. Ele tem conseguido evitar ser preso por violência doméstica? (por exemplo, ele não deixou você apresentar queixa; quando a polícia foi chamada, ele os convenceu de que tudo estava bem e que não tinha acontecido nada; ele fugiu antes da polícia chegar?). | | |
| ____ | 8. Você tem um filho [ou uma filha] que não é dele? | | |
| ____ | 9. Alguma vez ele forçou você a fazer sexo quando você não queria? | | |
| ____ | 10. Ele tenta estrangular ou esganar você? | | |
|  |  | 10a. (Se sim, ele fez isto mais de uma vez ou fez você desmaiar ou ficar tonta e/ou com a visão turva escureceu a vista? Marque aquí ___). | |
| ____ | 11. Ele usa alguma droga ilegal? Por drogas quero dizer “estimulatnes”, anfetaminas, metanfetamina (cristal, *glass*, bola), cocaína ou outras. | | |
| ____ | 12. Ele bebe ou tem problemas com bebidas? (é alcoólatra) | | |
| ____ | 13. Ele controla a maioria ou todas as coisas que você faz? Por exemplo: ele diz quem pode ser sua amiga, quando você pode ver a sua família, quanto dinheiro pode gastar ou quando pode usar o carro? | | |
|  | (Se ele tenta controlar você, mas você não deixa, assinale aqui: ___). | | |
| ____ | 14. Ele violentamente e constantamente tem ciúmes de você? (Por exemplo, ele diz: “Se você não for minha, não vai ser de mais ninguém”). | | |
| ____ | 15. Ele bateu ou machucou você alguma vez enquanto estava grávida? | | |
|  | (Se nunca esteve grávida dele, assinale aqui:___). | | |
| ____ | 16. Ele ameaça ou alguma vez tentou se matar? | | |
| ____ | 17. Ele ameaça machucar os seus filhos? | | |
| ____ | 18. Você acredita que ele é capaz de te matar? | | |
| ____ | 19. Ele segue ou vigia você, deixa mensagens ameaçadoras, quebra as suas coisas, ou liga para você quando você não quer falar com ele? | | |
| ____ | 20. Você ameaça ou alguma vez tentou se matar? | | |
| ____ | Número total de respostas “Sim”. | | |

**Obrigada. Por favor conversa com sua enfermeira**, **advogado, ou conselheiro sobre o que significa a Avaliação de Risco em sua situação.**
